# Adiposity in preadolescent children: Associations with cardiorespiratory fitness

**DOI:** 10.1371/journal.pone.0275982

**Published:** 2022-10-26

**Authors:** Nicholas Castro, Lauren C. Bates, Gabriel Zieff, Patricia Pagan Lassalle, James Faulkner, Sally Lark, Michael Hamlin, Paula Skidmore, T. Leigh Signal, Michelle A. Williams, Simon Higgins, Lee Stoner

**Affiliations:** 1 School of Health and Applied Human Sciences, University of North Carolina at Wilmington, Wilmington, NC, United States of America; 2 Department of Exercise and Sport Science, University of North Carolina at Chapel Hill, Chapel Hill, NC, United States of America; 3 School of Sport, Health, and Community, University of Winchester, Winchester, United Kingdom; 4 School of Sport, Exercise, and Nutrition, Massey University, Wellington, New Zealand; 5 Department of Tourism, Sport and Society, Lincoln University, Christchurch, New Zealand; 6 Department of Medicine, University of Otago, Dunedin, New Zealand; 7 Sleep-Wake Research Centre, Massey University, Wellington, New Zealand; 8 Department of Epidemiology, Harvard T. H. Chan School of Public Health, Boston, MA, United States of America; Universidade Estadual Paulista Julio de Mesquita Filho - Campus de Bauru, BRAZIL

## Abstract

Lifestyle factors contribute to childhood obesity risk, however it is unclear which lifestyle factors are most strongly associated with childhood obesity. The purpose of this cross-sectional study was to simultaneously investigate the associations among dietary patterns, activity behaviors, and physical fitness with adiposity (body fat %, fat mass, body mass index [BMI], and waist to hip ratio) in preadolescent children. Preadolescent children (N = 392, 50% female, age: 9.5 ± 1.1year, BMI: 17.9 ± 3.3 kg/m^2^) were recruited. Body fat (%) and fat mass (kg) were measured with bioelectrical impedance analysis. Cardiorespiratory fitness (VO_2_ max), muscular strength (hand-grip strength), activity, sleep, and dietary pattern was assessed. Multivariable analysis revealed that cardiorespiratory fitness associated most strongly with all four indicators of adiposity (body fat (%) (β = -0.2; *p* < .001), fat mass (β = -0.2; *p* < .001), BMI (β = -0.1; *p* < .001) and waist to hip ratio (β = -0.2; *p* < .001). Additionally, fruit and vegetable consumption patterns were associated with body fat percentage, but the association was negligible (β = 0.1; p = 0.015). Therefore, future interventions should aim to promote the use of cardiorespiratory fitness as a means of reducing the obesity epidemic in children.

## Introduction

Obese children are at heightened risk for developing early-onset cardiometabolic diseases such as type 2 diabetes and hypertension [1]. Considering the global rise in childhood obesity, this is a major public health concern [2]. For example, in New Zealand an estimated 1 in 10 children are overweight or obese [3] and prevalence has increased from 8% in 2006–07 to 14.9% in 2017–2018 [4]. Emerging evidence suggests the rise in obesity is likely attributed to lifestyle factors such as unhealthy dietary pattern and activity (physical activity [PA], sedentary [SB], sleep) behaviors [5] coupled with poor physical fitness (low cardiorespiratory fitness [CRF] and low muscular strength). However, it is unclear how simultaneous associations of lifestyle factors and adiposity present in preadolescent children. Identifying the lifestyle factors from a comprehensive investigation of potential risk factors that most strongly associated with childhood obesity is crucial for informing the design of public health interventions.

Obesity is a multidimensional disease in children (5–12 years old). Lifestyle factors such as insufficient daily PA [6, 7], high SB [6, 7], low physical fitness [8–10], poor sleep [11–13], and unhealthy dietary patterns [14] all likely contribute in both independent and interactive ways. Lifestyle factors interact with one another [15], where one activity behavior influences time spent, or not spent, in the other behaviors. For example, when children engage in SB (any waking behavior in a seated or reclined posture with low energy expenditure <1.5 metabolic equivalents [16] such as screen time, they are not only being physically inactive, but may also be snacking on unhealthy foods [15]. While our data is not sufficient to explore this, in the aforementioned theoretical example SB is not the only activity increasing obesity risk, instead SB is acting in combination with reduced PA and unhealthy dietary patterns to increase obesity risk.

The purpose of this cross-sectional study was to investigate the associations among lifestyle factors (PA, SB, sleep [duration, social jetlag, disturbance], physical fitness (CRF and muscular strength) and dietary patterns with adiposity in preadolescent children.

## Methodology

This observational study was carried out in accordance with STROBE (Strengthening the Reporting of Observational Studies in Epidemiology) guidelines [17]. The methodology was prospectively detailed in Castro et al. [18].

### Study design and participants

Children aged 8 to 10 years were randomly sampled from schools in three major cities across New Zealand (Wellington, Christchurch, and Dunedin). At invited schools, all children were eligible to participate unless they had an orthopedic injury or surgery that prohibited full physical function within the previous 4 weeks or were currently prescribed any cardiovascular medications. Parental or guardian consent and child assent were obtained prior to participation in accordance with the requirements of the New Zealand Health Disability Ethics Committee (14/CEN/83) and registered with the Australia and New Zealand Clinical Trial Registry (ACTRN12614000433606).

Data for this study were collected as part of a larger cross-sectional study [18]. The measurements detailed in this article were taken between 9 AM and 12 PM at the child’s school. Children were asked to fast for 3 hours and to refrain from exercise for 24 hours prior to assessment. Within 7 days of the in-person assessments described below PA/SB, dietary patterns, sleep habits, and demographic data were collected using a questionnaire. The questionnaires were jointly completed at home by the primary caregiver and participant using an online survey.

### Outcome measures

The four outcome measures included: body fat (%), fat mass index (FMI), body mass index (BMI), and waist to hip ratio (WHR).

#### Anthropometric

To calculate the anthropometric indices height, weight, and waist and hip circumference were measured. Height and weight were measured to the nearest 0.1 decimal, using a calibrated portable stadiometer (Seca 213, Hamburg, Germany) and a calibrated portable scale (Seca 813, Hamburg, Germany), respectively, with shoes and socks removed and head in the Frankfort plane. Using nonelastic tape (Seca 203, Hamburg, Germany), waist and hip circumference were measured to the nearest 0.1 cm according to standard practice to measure WHR [13]. For each assessment, participants were measured twice, and the average was recorded (unless the two measurements were more than 0.5 cm apart; then a third measurement was taken and the average of the three was recorded) [13]. Age and sex-specific BMI *z*-scores were calculated using the World Health Organization growth guidelines [19]. BMI values were categorized using the International Obesity Task Force’s sex and age-dependent cutoff points [20].

#### Body fat

Body fat (%) and fat mass (kg) were measured via multifrequency body impedance analysis (BodyStat Quadscan 4000, Isle of Man, UK). The instrument was calibrated in accordance with the manufacturer’s instructions, and measurements were conducted according to standardized procedures [21]. FMI was calculated by dividing fat mass (kg) by height squared (m^2^) [22].

### Independent variables

The ten behavioral variables measured included: PA, SB, sleep (duration, social jetlag, disturbance), dietary patterns (processed foods, fruits and vegetables, breakfast food), and physical fitness (CRF, muscular strength).

#### Physical activity and sedentary behavior

The Youth Physical Activity Questionnaire (YPAQ) was used to measure PA and SB [23]. To determine how many minutes a day each participant was active and sedentary, participants and their caregiver were asked to jointly complete the 47-item YPAQ. The YPAQ assessed the frequency, duration, and type of PA and SB the participant took part in 7 days prior to data collection [24, 25]. Frequency and duration were used to calculate the total number of active and sedentary minutes on a day-to-day basis, giving each participant a daily average and weekly total of active and sedentary minutes. Types of activities were utilized to classify actions as active movements or SB. For example, playing rugby, walking to school, or skipping were considered being active, whereas reading, watching television, and doing homework were considered being sedentary.

#### Sleep

Sleep duration, social jetlag (the discrepancy between an individual’s circadian clock and social rhythm) [13], and sleep disturbances were recorded to evaluate sleep. To determine average sleep duration, the participant’s caregiver(s) was/were asked to note what time their child usually went to bed and what time they usually got up on both school days and weekend days. Single items of habitual school/weekday sleep show reasonable concurrent validity with actigraphy and diary data [26]. Average sleep duration was calculated using a ratio of 5 weekdays to 2 weekend days. Social jetlag was calculated as the absolute difference between the midpoints of sleep on weekdays versus weekend days [27]. Sleep disturbances were recorded using the 33-item Children’s Sleep Habit Questionnaire (CSHQ), which demonstrates adequate internal consistency, acceptable test-retest reliability, and discriminant validity [28]. The 33 questions were answered on a 7-point Likert scale from 7 (*always*) to 0 (*never*), with higher scores indicative of greater sleep disturbance. The CSHQ includes eight subscales that align with the key sleep complaints relevant for this age group: bedtime resistance, sleep onset delay, sleep duration, sleep anxiety, night waking, parasomnias, sleep-disordered breathing, and daytime sleepiness. A Total Sleep Disturbances score was calculated as the sum of all CSHQ scored questions, with a potential range of 33 to 99. A Total Sleep Disturbances score > 41 was used to indicate significant pediatric sleep disturbance, as this cutoff point has been shown to accurately identify 80% of children with a clinically diagnosed sleep disorder [28]. For this study, only the Total Sleep Disturbances score was analyzed [28].

#### Dietary patterns

The Physical activity, Exercise, Diet And Lifestyle Study (PEADALS)-Food Frequency Questionnaire (FFQ) was used to assess dietary patterns of participants [14]. The 28-item PEDALS-FFQ has been validated in this age group and shows acceptable reliability and validity [29]. In this study, these 28 items were aggregated into 21 groups, and principal components analysis (PCA) was conducted to identify components/patterns from these 21 food groups [11, 29]. PCA restructures large data samples into new combined variables called principal components [30]. The principal components account for variation in the sample, enabling the dietary data to be captured with fewer variables. Determining the number of components/patterns to be retained was based on the eigenvalues > 1, identification of the point of inflection in the scree plot, and the interpretability of factors within components/patterns [31]. Three dietary components/patterns were identified including i.) processed food, ii.) fruit and vegetables, and iii.) breakfast food.

#### Physical fitness

CRF (VO_2_ max) was estimated via the 20-meter multistage fitness test (20-MST). The 20-MST has been found to be valid and noninvasive, is portable and space efficient, and is popular in school settings as many students can be tested simultaneously [32–34]. Participants ran in groups of 12–15 children continuously between two lines (20 meters apart) at a running speed (indicated by beep signal) that started at 8 km/hour in the first stage and then incrementally increased pace each minute (1km/hour for the first minute and by 0.5 km/hour in each minute stage following). The final stage was determined when the participants failed to reach the line before the signal on two consecutive occasions (beep signals) or when the child voluntarily withdrew. VO_2_ max was estimated using the regression equation established by Hamlin et al., [33] which has been previously validated on New Zealand children: [VO_2_ max (ml/kg) = 42.18 + (0.009 x 20 m) + (-0.1762 x body fat%) + (-0.4091 x maturity)]. Anthropometric measures were used to calculate maturity in accordance with Mirwald et al., 2002 [35] using height, sitting height, leg length, chronological age, and their interactions. In accordance with the Cooper Institute (2014) FitnessGram^*®*^ cutoff points (beep signal), a “healthy CRF zone” (high) was reported if females achieved a VO_2_ max equal to or greater than 39 mL/kg/min and if boys achieved equal to or greater than 42 mL/kg/min [36, 37]. A VO_2_ max below those cutoff points was categorized as “needs improvement fitness zone” (low) for both sexes [37].

Muscular strength (isometric handgrip measured in kg) was assessed using a handgrip dynamometer (Camry, EH101). This method is rapid, noninvasive, simple to use, inexpensive, and of minimal risk [38]. The participants were seated with shoulders adducted and neutrally rotated, elbow flexed to 90 degrees, and wrist in a neutral position. Each participant was given three attempts with each hand, alternating hands, and with a minute recovery time between each attempt. The highest score for each hand was recorded for analysis.

### Covariate measurements

Demographic data collected included participants’ date of birth, age, sex, ethnicity, and school address. The ethnicity data were applied to categorize participants into four classifications: (a) New Zealand European and Others (NZEO), (b) Māori, (c) Pacific, and (d) not specified. The majority of schools in New Zealand are publicly funded and classified by the predominance of students attending, giving the schools a decile classification. Schools with a greater proportion of student from low socio-economic communities (decile 1) receive more funding than those fewer students from low socio-economic communities (decile 10). To achieve a cohort representing different levels of socio-economic position (SEP), schools within the selected cities were stratified as low (1–5) or high (6–10) decile and then randomly sampled. Statistical models were adjusted for sex, ethnicity, age, and school decile as an index of SEP.

### Statistical analysis

The corresponding author had full access to the data in the study and was responsible for the integrity of the data set and the data analysis. Anonymized data will be shared upon reasonable request. Only participants who had complete data for each independent and outcome variable were included in the analyses. Statistical analyses were performed using R Statistical Software, version 4.0.0. Raw data are presented as mean (standard deviation) and regression outcomes as unstandardized (b) and standardized (β) betas (effect sizes). Using the β, the effect was adjudicated as trivial (<0.2), small (0.2–0.5), moderate (0.5–0.8), or large (≥0.8). Additionally, point (two-sided p-value) and interval (95% confidence interval) estimates of statistical significance are presented, with two-sided p-values of <0.05.

Linear mixed-effects models, with children nested within schools, were used to identify relationships among the independent- (physical fitness and lifestyle factors) and outcome- (adiposity) variables [39]. Model 1: univariable analysis, in which each independent variable was regressed against each adiposity outcome. For each independent variable, linearity was explored by specifying the quadratic term. In the event of non-linearity, to minimize collinearity the independent variable was centered and then used to create the quadratic term. An independent variable was omitted from Model 2 if it did not significantly associate at alpha < 0.10 with one of the adiposity outcomes. Model 2: unadjusted multivariable analyses, in which all significant independent variables were regressed against each adiposity outcome. Model 3: the multivariable models (i.e., Model 2) were adjusted for sex, ethnicity, age, and school decile. All regression models were assessed by examination of the model residuals plotted against their normal scores. The assumptions of normality and homoscedasticity were assessed via visual inspection of the frequency and residual distributions, respectively. To test for multicollinearity, variance inflation factors were compared to the recommended cut-point of 10.

## Results

### Participants

**Fig 1:**
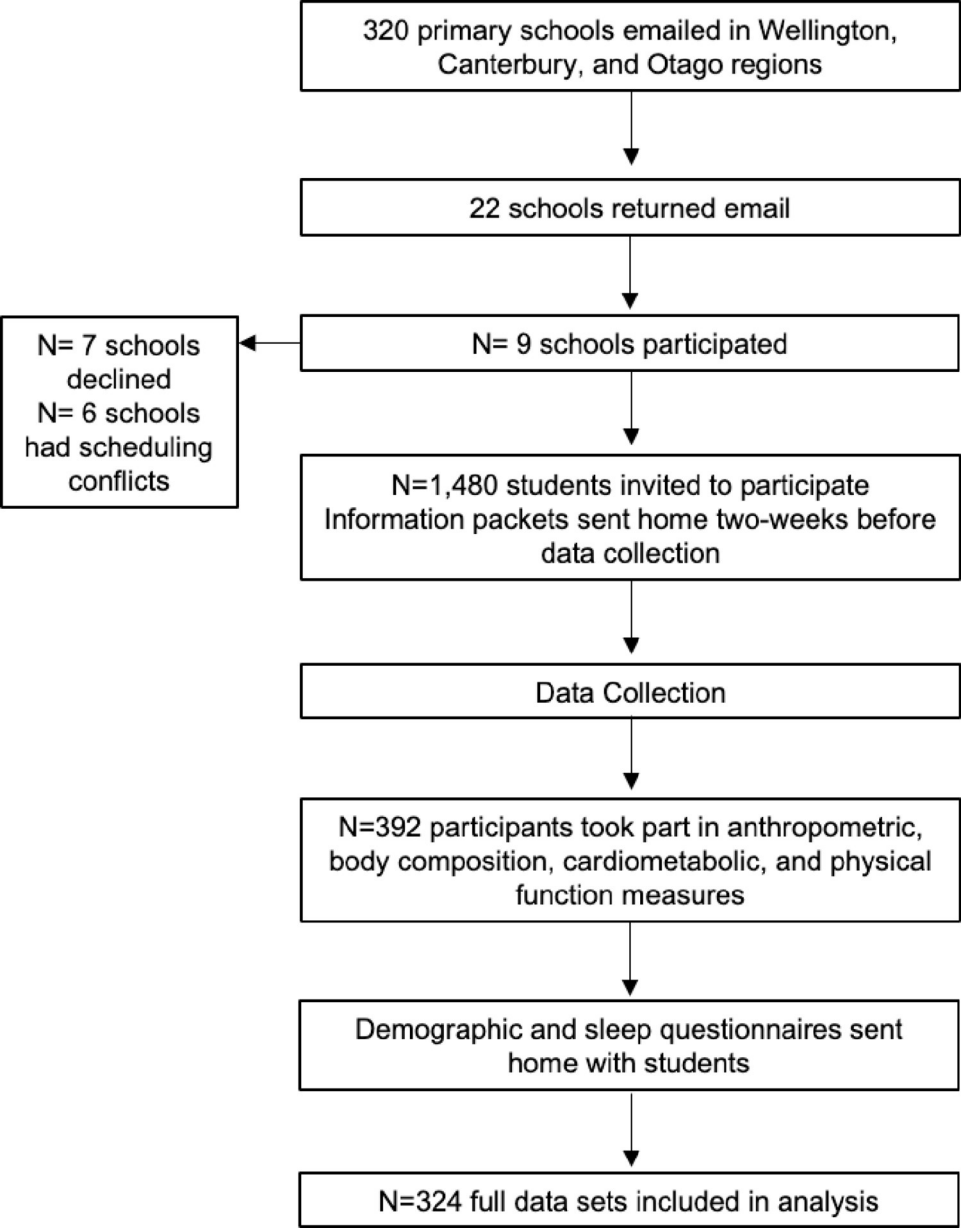
Study design and protocol. Includes information regarding recruitment, data collection, and analysis timeframe.

Of the 392 participants who took part in the study, only 324 participants had complete data sets (Table 1, Fig 1). Participant characteristics including age, anthropometrics, ethnicity, school decile, obesity classification, CRF classification, PA, SB, and sleep are reported (Table 1).

**Table 1:** Summary of participant characteristics.

	Total (N = 324)	
	Mean or *n*	SD/%
Age (years)	9.56	1.14
Ethnicity		
NZEO	265	82
Māori	37	11
Pacific	18	6
NR	4	1
School year		
4	82	21
5	114	29
6	127	32
7	69	18
School Decile (SEP)		
Low (≤5)	163	50
High (>5)	161	50
Adiposity		
Body Fat (%)	19.9	9.39
FMI (kg/m^2^)	3.61	2.37
WHR	0.84	0.05
BMI (kg/m^2^)	17.9	3.25
Overweight	89	27
Non-Overweight	235	73
Physical Activity & Sedentary Behavior		
Physical Activity (min/day)	166	136
Sedentary (min/day)	284	208
Cardiorespiratory Fitness		
VO_2_max (ml•kg-1•min-1)	42.9	4.36
Low	150	45
High	174	52
Sleep		
Average Sleep Duration (hours)	10	1
Social Jetlag (hours)	1	1
Sleep Disturbances	40.3	5.94

Abbreviations: SEP, socio-economic position; NZEO, New Zealand European or other; NR not recorded; FMI, fat mass index; WHR, waist-to-hip ratio; BMI, body mass index; kg/m^2^: kilogram/meters; VO_2_max: maximum volume of oxygen uptake; ml•kg-1•min-1: milliliters/kilogram/minute.

Participant characteristics including demographics summarized in Table 1. Data reported as the mean (or N) with standard deviation.

### Univariate models

**Fig 2:**
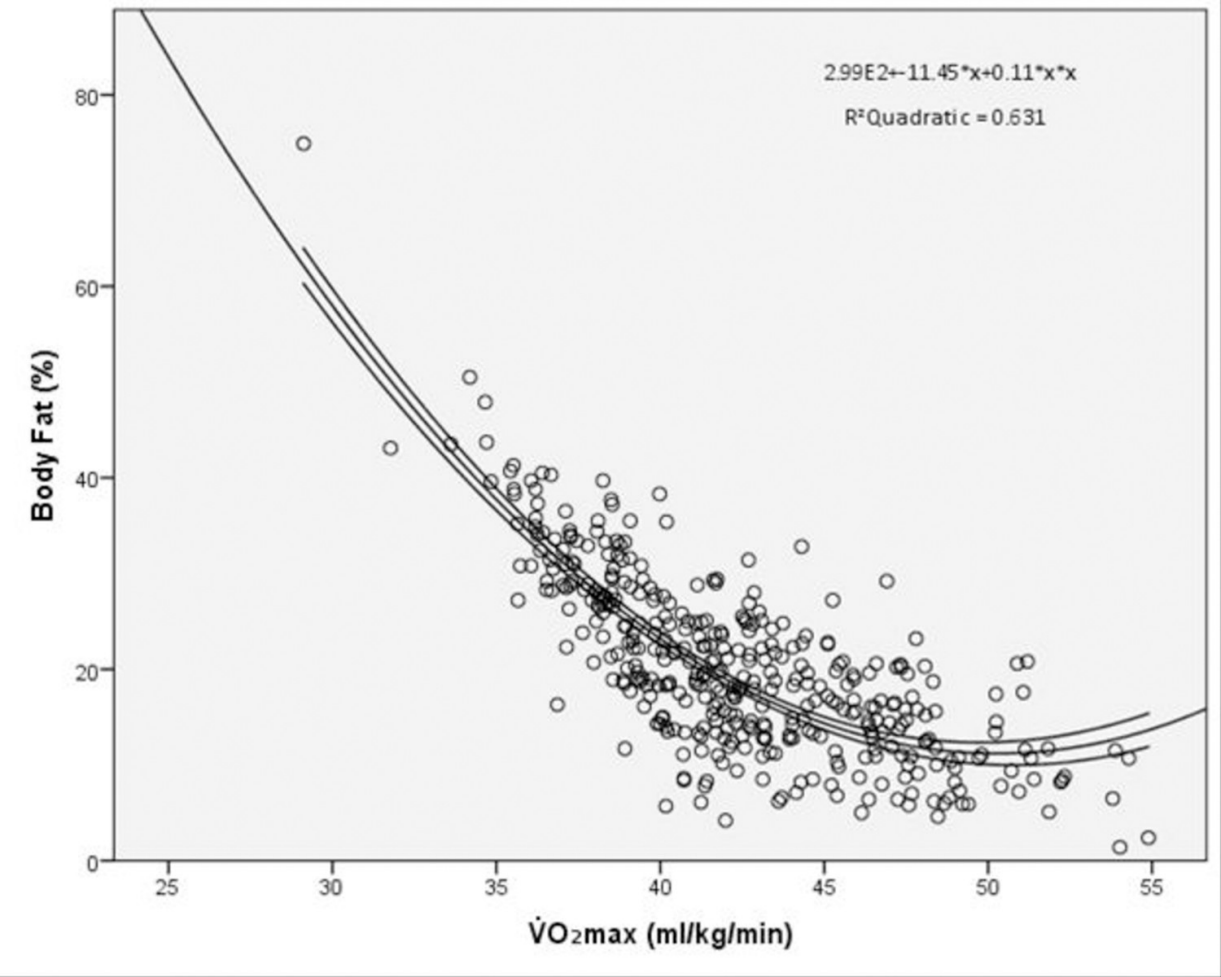
VO_2_ max and body fat percentage linear quadratic and cubic analysis. Figure displays the nonlinear relationship between body fat (%) and VO_2_ max.

Model 1, Table 2 presents the independent variables that significantly associated with at least one of the adiposity outcomes. PA, SB, and handgrip strength did not significantly associate with one of the adiposity outcomes and were omitted from multivariable analysis. The association of body fat (%) with VO_2_ max was non-linear, therefore VO_2_ max and the associated non-linear/polynomial (VO_2_ max_Poly_) were used to account for non-linearity (Fig 2).

**Table 2:** Linear association between adiposity and lifestyle factors.

	Univariable					Multivariable									
	Model 1					Model 2 (school adjusted)					Model 3 (adjusted)				
	*β*	*b*	95%LCI	95%UCI	*P*	*β*	*b*	95%LCI	95%UCI	*P*	*β*	*b*	95%LCI	95%UCI	*P*
Body Fat (%)															
${\dot{V}O}_{2}\max$	-0.8	-1.6	-1.8	-1.5	**< .001**	-0.2	-1.6	-1.8	-1.5	**< .001**	-0.2	-1.6	-1.8	-1.5	**< .001**
${\dot{V}O}_{2}\max$ Poly	0.3	0.1	0.1	0.1	**< .001**	0.3	0.1	0.1	0.1	**< .001**	0.0	0.1	0.1	0.1	**< .001**
Sleep Duration	0.0	0.3	-0.8	1.5	0.595	0.1	0.6	-0.1	1.4	0.103	0.1	0.7	-0.1	1.4	0.085
Social Jetlag	0.1	2.6	0.8	4.4	**0.005**	0.0	0.5	-0.7	1.7	0.421	0.1	0.4	-0.8	1.6	0.518
Sleep Disorders	0.1	0.2	0.0	0.4	**0.013**	0.0	0.1	0.0	0.2	0.182	0.0	0.1	0.0	0.2	0.189
Processed Foods	0.1	0.5	-0.1	1.1	0.088	0.0	0.1	-0.3	0.4	0.766	0.0	0.1	-0.3	0.4	0.725
Fruits/Vegetables	-0.1	-0.3	-1.0	0.3	0.279	0.1	0.5	0.1	0.9	**0.012**	0.1	0.5	0.1	0.9	**0.015**
Breakfast Foods	-0.1	-0.9	-1.7	-0.2	**0.018**	0.0	-0.4	-0.9	0.1	0.154	0.0	-0.4	-0.9	0.1	0.150
Fat Mass Index (kg/m^2^)															
${\dot{V}O}_{2}\max$	-0.8	-0.4	-0.4	-0.4	**< .001**	-0.2	-0.4	-0.4	-0.4	**< .001**	-0.2	-0.4	-0.5	-0.4	**< .001**
${\dot{V}O}_{2}\max$ Poly	0.4	0.0	0.0	0.0	**< .001**	0.4	0.0	0.0	0.0	**< .001**	0.0	0.0	0.0	0.0	**< .001**
Sleep Duration	0.0	-0.1	-0.4	0.2	0.533	0.0	0.0	-0.2	0.2	0.945	0.0	0.1	-0.1	0.3	0.496
Sleep Disorders	0.0	0.1	0.0	0.1	**0.006**	0.0	0.1	-0.2	0.4	0.624	0.0	0.0	-0.2	0.3	0.767
Social Jetlag	0.3	0.7	0.2	1.1	**0.004**	0.0	0.0	0.0	0.0	0.143	0.0	0.0	0.0	0.0	0.095
Processed Foods	0.1	0.1	0.0	0.3	0.078	0.0	0.0	-0.1	0.1	0.948	0.0	0.0	-0.1	0.1	0.795
Fruits/Vegetables	-0.1	-0.2	-0.3	0.0	0.067	0.1	0.1	0.0	0.2	0.133	0.0	0.1	0.0	0.2	0.102
Breakfast Foods	-0.1	-0.2	-0.4	0.0	**0.027**	0.0	-0.1	-0.2	0.0	0.170	0.0	-0.1	-0.2	0.0	0.227
Body Mass Index (kg/m^2^)															
${\dot{V}O}_{2}\max$	-0.5	-0.1	-0.2	-0.1	**< .001**	-0.1	-0.1	-0.2	-0.1	**< .001**	-0.1	-0.2	-0.2	-0.1	**< .001**
${\dot{V}O}_{2}\max$ Poly	0.3	0.0	0.0	0.0	**< .001**	0.3	0.0	0.0	0.0	**< .001**	0.0	0.0	0.0	0.0	**< .001**
Sleep Duration	0.0	0.0	-0.2	0.1	0.556	0.0	0.0	-0.2	0.1	0.801	0.0	0.0	-0.1	0.2	0.672
Sleep Disorders	0.0	0.0	0.0	0.0	0.061	0.0	0.1	-0.1	0.3	0.468	0.1	0.1	-0.1	0.3	0.382
Social Jetlag	0.2	0.3	0.0	0.5	**0.037**	0.0	0.0	0.0	0.0	0.522	0.0	0.0	0.0	0.0	0.337
Processed Foods	0.0	0.0	-0.1	0.1	0.890	-0.1	0.0	-0.1	0.0	0.296	0.0	-0.1	-0.1	0.0	0.116
Fruits/Vegetables	-0.1	-0.1	-0.2	0.0	**0.038**	0.0	0.0	-0.1	0.1	0.896	0.0	0.0	-0.1	0.1	0.795
Breakfast Foods	-0.1	-0.1	-0.2	0.0	**0.046**	-0.1	-0.1	-0.1	0.0	0.241	0.0	0.0	-0.1	0.1	0.429
Waist: Hip															
${\dot{V}O}_{2}\max$	0.0	0.0	0.0	0.0	**0.014**	0.0	0.0	0.0	0.0	**0.025**	-0.2	0.0	0.0	0.0	**< .001**
${\dot{V}O}_{2}\max$ Poly	0.0	0.0	0.0	0.0	**0.019**	0.0	0.0	0.0	0.0	**0.028**	0.1	0.0	0.0	0.0	0.064
Sleep Duration	-0.1	0.0	0.0	0.0	0.220	-0.1	0.0	0.0	0.0	0.245	0.0	0.0	0.0	0.0	0.384
Sleep Disorders	0.0	0.0	0.0	0.0	0.948	0.1	0.0	0.0	0.0	0.354	0.1	0.0	0.0	0.0	0.229
Social Jetlag	0.1	0.0	0.0	0.0	0.149	0.0	0.0	0.0	0.0	0.619	0.0	0.0	0.0	0.0	0.782
Processed Foods	0.0	0.0	0.0	0.0	0.808	0.0	0.0	0.0	0.0	0.693	0.0	0.0	0.0	0.0	0.399
Fruits/Vegetables	0.0	0.0	0.0	0.0	0.479	0.0	0.0	0.0	0.0	0.881	0.0	0.0	0.0	0.0	0.826
Breakfast Foods	0.0	0.0	0.0	0.0	0.862	0.0	0.0	0.0	0.0	0.963	0.0	0.0	0.0	0.0	0.782

Model 1: univariable.

Model 2: school only.

Model 3: gender, ethnicity, age, decile.

Linear association between adiposity and lifestyle factors displayed with significant associations for each model bolded. Abbreviations: UCI: upper confidence interval; LCI: lower confidence interval; kg/m^2^: kilogram/meters; VO_2_max: maximum volume of oxygen uptake; ml•kg-1•min-1: milliliters/kilogram/minute.

### Multivariable models

For Model 2, unadjusted multivariable analyses, VO_2_ max was associated with all four adiposity measures: body fat (%), FMI, BMI, and WHR (β = -0.2, -0.2, -0.1, 0.0, respectively; all *p* < 0.05, Table 2). Additionally, fruit and vegetable consumption were associated with body fat (%) (β = 0.1, *p* = 0.012). For Model 3, which adjusted for sex, ethnicity, age, and school decile, VO_2_ max remained associated with all four adiposity measures: body fat (%), FMI, BMI, and WHR (β = -0.2, -0.2, -0.1, -0.2, respectively; all *p* < 0.001, Table 2), and fruit and vegetable consumption remained associated with body fat (%) (β = 0.1, *p* = 0.015, Table 2).

## Discussion

The purpose of this study was to investigate the associations among activity behaviors including physical fitness, PA, SB, dietary patterns, and sleep with adiposity in preadolescent children. Following adjustments for potential confounders, this study clearly indicated that CRF, as measured VO_2_ max estimated via the 20-meter multistage fitness test, was associated with high body fat percentage, greater BMI, greater FMI, and high WHR. Fruit and vegetable consumption independently associated with body fat percentage. The effect of the association between fruit and vegetable consumption and body fat percentage was not in the expected direction however, it should be noted the effect as negligible. Our findings suggest that increases in CRF may result in lower levels of adiposity outcomes, and thereby obesity risk in preadolescent children.

### Limitations and strengths of the study

This study had several potential limitations which are important to consider prior to contextualizing the results. First, this was a cross-sectional study whereas further longitudinal research is required to better determine the potential causal relationships between activity behaviors with adiposity. However, this initial investigation was necessary prior to allotting time and resources into costly trials. Second, data collection was conducted at primary schools in group settings; therefore, the noise, facility limitations (e.g., secluded space, tinted windows, and private room), distractions, interruptions, and weather could not be controlled or measured [40]. Lastly, this study investigated New Zealand-based preadolescents, which may limit the generalizability of the findings to preadolescent children globally. For instance, dietary intake (Mediterranean diet vs. Western diet), types of PA (rugby vs. American football), and social ecological factors may affect a child’s physiology differently, so further research is required to determine if these findings would be consistently associated with preadolescent outcomes globally. However, a considerable strength of this study was the large and diverse group of New Zealand-based preadolescents included in the research from various parts of the country. Similar studies have shown comparable results to this study, but most of those studies analyzed each factor independently with adiposity [10, 12, 41–44].

### Cardiorespiratory fitness

Of the lifestyle and physical fitness factors measured, only CRF associated solely with all four estimates of adiposity (body fat [%], FMI, BMI, and WHR). However, it should be recognized that CRF was nonlinearly associated with body fat percentage. Beyond ~42 ml/kg/min, an increase in VO_2_ max did not correspond with change in body fat percentage. This suggests that it may be particularly important to focus on improving CRF in children with a VO_2_ max below 42 ml/kg/min. Compared to normative data [45], a VO_2_ max of 42 ml/kg/min in children is classified as “fair” [46]. Therefore, “fair”, “poor”, or “very poor” VO_2_ max classification [46] is associated with higher body fat percentage. These data are similar to other studies which have also demonstrated association between lower VO_2_ max and adiposity [47–50], however, the present study provides continuous data to better elucidate the non-linear association between VO_2_ max and adiposity, which is lost when VO_2_ max is dichotomized into classifications. Collectively, children and adolescents with low levels of CRF are at greater risk of myocardial infarction, CVD, and sustaining lower than average physical fitness levels in adulthood [51]. Additionally, it is important to note that VO_2_ max classification differs by biological sex and age (i.e., puberty stage). Associations of maturation, sex, and body fat with VO_2_ max have been previously demonstrated [52] where VO_2_ max is higher in males. In the present study despite the same weight (kg) between males and females, there are differences in body fat percentage between the biological sexes. At the onset of puberty, females tend to have higher adiposity indicating classifications for outcomes such as VO_2_ max should account for biological sex.

### Dietary pattern and sleep

In this study, fully adjusted multivariable analyses indicated no association between any sleep variable with adiposity, and no association of any dietary pattern with BMI, FMI, and WHR. However, there was a small, although statistically significant (0.1, *p* = 0.015) association of the fruit and vegetables dietary pattern with body fat percentage in preadolescent children. More fruit and vegetable consumption was associated with a higher body fat percentage, which challenges our hypothesis that fruit and vegetable consumption would be associated with a lower body fat percentage [53]. This contrary association is likely due to collinearity with an additional variable; we should also emphasize that the association was trivial. Additionally, it may be that individuals with higher body fat percentage likely consume more calories, and thus have a higher intake of every type of food including fruit and vegetables. Further, we should emphasize that our findings should not be interpreted to indicate that diet and sleep are insignificant to adiposity. For example, sleep has been associated with other health behaviors, including diet [7, 54], and both sleep and diet may be determinants of optimal CRF. While beyond the scope of the current paper, further research is warranted to elucidate the likely complex relationships between sleep, diet and activity behaviors with CRF and adiposity. It is important to note that while diet, SB, sleep, and PA are undoubtably important to the health and development of a child, our findings suggest that CRF is a particularly useful systems physiology target for health-based interventions in this population.

### Implications

CRF had the strongest association with adiposity in preadolescent children. Devoting resources towards prevention and intervention strategies that target improving and maintaining CRF could be one of the essential components for addressing the childhood obesity epidemic, and subsequently making an impact on the deteriorating health and wellness of preadolescent children. CRF is extremely important in potentially mitigating adiposity in preadolescents and should be targeted by improving PA levels. In adults, PA contributes to about 30% of VO_2_ max whereas genetics controls the remaining 70% [55]. In comparison, PA contributes to a moderate amount of VO_2_ max in children [56]. Furthermore, estimating CRF with a shuttle run test is a feasible and reliable measurement schools can use to track CRF as children age. Therefore, participation in PA that increases heart rate, reducing SB, and engaging in sport/physical education classes at school should be at the forefront of pediatric health because they are associated with improving CRF.

## Conclusions

CRF is an especially important target for preventing adiposity in preadolescent children. Lifestyle factors inter-relate with one another and additional factors such as unhealthy dietary patterns, poor sleep (i.e., high sleep disturbance or social jetlag), and poor activity behaviors (low PA and high SB) are also important to consider when attempting to mitigate adiposity risk. These preliminary findings suggest that CRF correlates most strongly with adiposity (all four adiposity estimators) and therefore support the development of preventative measures (i.e., policy and access to physical activity in school) to increase CRF to mitigate adiposity risk in preadolescent children. Therefore, future interventions and public health guidelines should strive to improve CRF in preadolescent children to prevent obesity.

## Supporting information

S1 Data (CSV)
